# Comparative Radioimmunotherapy of Experimental Melanoma with Novel Humanized Antibody to Melanin Labeled with 213Bismuth and 177Lutetium

**DOI:** 10.3390/pharmaceutics11070348

**Published:** 2019-07-18

**Authors:** Kevin J. H. Allen, Rubin Jiao, Mackenzie E. Malo, Connor Frank, Darrell R. Fisher, David Rickles, Ekaterina Dadachova

**Affiliations:** 1 College of Pharmacy and Nutrition, University of Saskatchewan, Saskatoon, SK S7N 5E5, Canada; 2 Versant Medical Physics and Radiation Safety, Richland, WA 99354, USA; 3 RadImmune Therapeutics, Tarrytown, NY 10591, USA

**Keywords:** radioimmunotherapy, humanized antibody, melanin, B16-F10 melanoma, 213Bismuth, 177Lutetium

## Abstract

Melanoma is a cancer with increasing incidence and there is a need for alternatives to immunotherapy within effective approaches to treatment of metastatic melanoma. We performed comparative radioimmunotherapy (RIT) of experimental B16-F10 melanoma with novel humanized IgG to melanin h8C3 labeled with a beta emitter, ^177^Lu, and an alpha-emitter, ^213^Bi, as well as biodistribution, microSPECT/CT imaging, and mouse and human dosimetry calculations. microSPECT/CT imaging showed that a humanized antibody that targets “free” melanin in the tumor microenvironment had high tumor uptake in B16F10 murine melanoma in C57Bl/6 mice, with little to no uptake in naturally melanized tissues. Extrapolation of the mouse dosimetry data to an adult human demonstrated that doses delivered to major organs and the whole body by ^177^Lu-h8C3 would be approximately two times higher than those delivered by ^213^Bi-h8C3, while the doses to the tumor would be almost similar. RIT results indicated that ^213^Bi-h8C3 was more effective in slowing down the tumor growth than ^177^Lu-h8C3, while both radiolabeled antibodies did not produce significant hematologic or systemic side effects. We concluded that h8C3 antibody labeled with ^213^Bi is a promising reagent for translation into a clinical trial in patients with metastatic melanoma.

## 1. Introduction

In the United States, melanoma, a cancer with growing frequency, is expected to affect roughly 96,480 new patients in 2019 with over 7230 projected to succumb to the disease [1]. While early stages of melanoma can be resected surgically, the highly metastatic nature of the later stages results in a very poor prognosis [2,3]. Ultimately, in patients with stage IV melanoma, the projected 10-year survival is only 9% [2,3]. As recently as 2011, treatment options for late stage melanoma (stage IV) were very limited and only offered small improvements, however, upon the approval of vemurafenib, a B-RAF inhibitor, initial results were promising in patients positive for the mutated B-RAF protein, roughly 40–60% of people diagnosed with melanoma [4]. However, the impressive early results of vemurafenib were generally short-term and were repeatedly followed by relapses [4]. Currently, research focused on re-establishing suppressed anti-tumor immunity has examined monoclonal antibody (mAb)-based interventions by targeting CTL antigen 4 (CTLA-4) [5,6] and programmed cell death protein 1 (PD-1) on T lymphocytes and its primary ligand (PD-L1) on tumor cells [7,8]. Treatment using ipilimumab, a CTLA-4 targeting antibody, shows restoration of tumor immunity at the priming phase and extends survival in some patients, conversely anti-PD-1/PD-L1 antibodies seem to affect the malignancy by returning immune function in the tumor microenvironment [7,8]. Unfortunately, only a small amount of patients respond well to the current immune therapies, coupled with very serious side effects and the large cost of these treatments [9,10] there is still a high demand for additional approaches to combat metastatic melanoma that can either stand alone or as a combination therapeutic treatment with available immunotherapies.

Targeted radionuclide therapy has been considered a promising approach to treatment of metastatic melanoma from the early days of mAbs introduction into research and clinical practice and included iodinated antibodies to high molecular weight melanoma associated antigen (HMW-MAA), pretargeted antibody-guided radioimmunotherapy (PAGRIT), intralesional and systemic alpha-radioimmunotherapy, as well as radiolabeled peptide analogues of melanocyte-stimulating hormone (MSH) receptor and small molecules such as radioiodinated benzamides reviewed in [11]. We have recently reported on the structure and preliminary encouraging in vivo evaluation of a murine IgG to melanin as a delivery vehicle in radioimmunotherapy (RIT) of melanoma. When radiolabeled with an alpha-emitter, 213Bismuth (^213^Bi), 8C3 mAb to melanin demonstrated significant therapeutic efficacy in B16-F10 lung “metastatic” murine melanoma, safety towards healthy melanin-containing tissues, and favorable comparison with the anti-CTLA4 antibody [12]. However, murine mAbs are not suitable for multiple administration to patients as they can cause a potentially dangerous immune response. For this reason, the humanization of 8C3 was performed [13]. Here we describe the comparative radioimmunotherapy of B16-F10 murine melanoma with ^213^Bi and 177Lutetium (^177^Lu) labeled humanized 8C3 (h8C3) mAb, its safety evaluation, and dosimetry results in mice and humans.

## 2. Materials and Methods 

Antibodies, reagents, and Radionuclides: murine and humanized 8C3 antibodies were produced by Aragen Bioscience (Morgan Hill, CA, USA). ^225^Ac for construction of the ^213^Bi/^225^Ac radionuclide generator was purchased from Oak Ridge National Laboratory, Oak Ridge, TN, USA. ^177^Lu in form of ^177^Lu chloride was acquired from Radiomedix (Houston, TX, USA), and ^111^In chloride from Nordion Canada (Ottawa, ON, Canada). Bifunctional chelating agent (BCA) *N*-[2-amino-3-(*p*-isothiocyanatophenyl)propyl]-*trans*-cyclohexane-1,2-diamine-*N*,*N*′,*N*″,*N*‴,*N*″″-pentaacetic acid (CHXA″) was procured from Macrocyclics, Dallas, TX, USA.

Conjugation of BCA CHXA″ to h8C3 antibody: 10X conjugation buffer (0.05 M Carbonate/Bicarbonate, 0.15 M NaCl, 5 mM EDTA, pH 8.6–8.7), 5 mL, was combined with 0.5 M EDTA, pH = 8.0 (0.5 mL) and was diluted to 50 mL in a 50 mL Falcon tube with deionized water to give the 1X buffer. An Amicon Ultra 0.5 mL centrifugal filter (30K MW cut off, Fisher, Hampton, NH, USA) was loaded with 2 mg of the h8C3 antibody. The antibody was exchanged into the above conjugation buffer by performing 10 × 0.4 mL washes using an Amicon concentrator in a refrigerated centrifuge at 4 °C. The antibody was recovered from the Amicon and 23.6 μL of 2 mg/mL CHXA″ solution in conjugation buffer was added to provide 5 fold molar excess of CHXA″ over the antibody. The reaction mixture was incubated at 37 °C for 1.5 h and then purified into 0.15 M ammonium acetate buffer, pH = 6.5–7.0, with 10 × 0.4 mL washes on Amicon concentrators in a refrigerated centrifuge at 4 °C. A Bradford assay was performed to determine protein recovery and concentration.

Radiolabeling of h8C3-CHXA″ conjugate with ^111^In, ^177^Lu, and ^213^Bi. The radiolabeling of h8C3-CHXA″ conjugate with ^111^In and ^177^Lu was performed to achieve the specific activity of 0.185 MBq/μg of the antibody. Then, 22.2 MBq of ^111^In or ^177^Lu chloride was added to 10 μL 0.15 M ammonium acetate buffer and added to a microcentrifuge tube containing 120 μg of the h8C3-CHXA″ conjugate in 0.15 M ammonium acetate buffer. The reaction mixture was incubated for 60 min at 37 °C, and then the reaction was quenched by the addition of 3 μL of 0.05 M EDTA solution. For radiolabeling with ^213^Bi at a specific activity of 0.185 MBq/μg of the antibody, ^213^Bi was eluted from a ^213^Bi/^225^Ac radionuclide generator with a 0.1 M HI solution. The pH of the solution was adjusted to 6.5 with 5 M ammonium acetate buffer prior to addition to the h8C3-CHXA″ and the reaction mixture was incubated for 15 min at 37 °C. The percentage of radiolabeling was measured by SG-iTLC using 0.15 M ammonium acetate buffer as the eluent (top containing unlabeled ^111^In, bottom containing protein conjugated ^111^In). SG-iTLCs were read on a Perkin Elmer 2470 Automatic Gamma Counter (Perkin Elmer, Waltham, MA, USA). ^111^In- and ^177^Lu-labeled h8C3 were used immediately with no need for further purification as greater than 95% labeling was routinely achieved while ^213^Bi-h8C3 was purified from HI on a disposable size exclusion filter giving radiochemical yields in excess of 98%.

Murine B16-F10 melanoma model. All animal studies were approved by the Animal Research Ethics Board of the University of Saskatchewan. For the imaging and therapy studies 6 weeks old C57BL6 female mice obtained from Charles River Laboratories (Wilmington, MA, USA) were injected subcutaneously with 5 × 10^5^ B16-F10 murine melanoma cells in Matrigel (Corning Inc, Corning, NY, USA) into the right flank.

microSPECT/CT imaging of B16-F10 melanoma tumor-bearing mice with ^111^In-h8C3. microSPECT/CT (micro single photon emission computer tomography/computer tomography) images were collected on a MILabs VECTor^4^ (The Netherlands) microSPECT/CT scanner and processed using the comprehensive image analysis software package PMOD (version 3.9, PMOD Technologies, Inc, Zürich, Switzerland). Imaging studies were conducted using 7.4 MBq ^111^In at a 0.185:1 MBq/mg specific activity with a CHXA″ conjugated h8C3. Two tumor-bearing mice were injected IV via tail vein and imaged in the prone position at 1, 4, 24, and 48 h post injection. SPECT data was collected for 20 min using an Extra Ultra High Sensitivity Mouse (XUHS-M) collimator for 20–350 keV range using spiral trajectories. All SPECT images were reconstructed using both 245 keV and 171 keV ^111^In gamma emissions on a 0.4 mm voxel grid with MILabs reconstruction software (MILabs, Utrecht, Netherlands). MIP images were used for visual representation of the tumor.

The biodistribution of ^111^In-h8C3. When the tumors in mice reached approximately 200 mm^3^, the mice were randomized into the groups of 5 animals and injected IV via the tail vein with 1.85 MBq (10 μg of mAb) of ^111^In-h8C3. At the pre-determined time points of 1, 2, 24, 48, and 72 h post-injection of the radiolabeled antibody the mice were humanely sacrificed, their major organs, blood, and tumors removed, weighted, and counted in Perkin Elmer 2470 Automatic Gamma Counter. As the melanin content in the skin of the tail can vary, we selected the most highly melanized portion to confirm the safety of our antibody. The results of the biodistribution were used for mouse and human dosimetry calculations for the proposed therapeutic radionuclides ^177^Lu and ^213^Bi.

Mouse dosimetry calculations for ^177^Lu-h8C3 and ^213^Bi-h8C3: using the decay corrected biodistribution data for ^111^In-h8C3, we calculated the radiation doses for ^177^Lu and ^213^Bi in mice by assuming either ^177^Lu and ^213^Bi in place of ^111^In. The mouse data was back-decay-corrected (percent administered activity per gram tissue) to obtain the effective data (related to actual counts) each for ^213^Bi (half-life is 45.6 min) and for ^177^Lu (half-life is 160 h). For each organ or tissue, the effective data points were plotted against sampling time, and linear least-squares regression analysis was used to obtain a best-fit single (or double) exponential function to the data, with best-fit equation parameters. The exponential function was integrated to obtain an estimate of the 37 kiloBecquerel-hours per 37 kiloBecquerel administered, represented by the area under the time-activity function, integrated to infinity (complete decay) for both the ^177^Lu and ^213^Bi cases. An absorbed fraction of 1.0 was assumed for all ^213^Bi emissions in the mouse organs and tissues (Table 1). The model values for ^177^Lu emissions for the fraction of energy emitted from the measured organ or tissue (Table 1) that deposits in the same organ or tissue were determined using the mouse model developed earlier by Miller et al. [14]. The equilibrium dose constants for ^213^Bi and ^177^Lu were obtained from [15]. For ^213^Bi, the equilibrium dose constant is 19.44 g cGy 37 kBq^−1^ h^−1^, and for ^177^Lu, the equilibrium dose constant is 0.315 g cGy 37 kBq^−1^ h^−1^. Knowing the equilibrium dose constant, the absorbed fraction of emitted beta energy, and the integral activity residing in the organ or tissue through complete decay, the absorbed dose in units of cGy (centigray) per 37 kilobecquerel (cGy/37 kBq) were calculated and administered (average dose and correlation coefficients).

Human dosimetry calculations for ^177^Lu-h8C3 and ^213^Bi-h8C3. To calculate the doses which would be delivered by ^213^Bi-h8C3 and ^177^Lu-h8C3 in a human, extrapolation from mouse data was performed. The extrapolation was performed by recalculating the residence times for the human model from the mouse model, and calculating the human doses using a MIRD schema implementing software OLINDA1.1 (Hermes Medical Solutions Inc. Greenville, NC, USA). The method assumes proportionality based on weight differences between species [16]:

(1) Rh=Rm(OhBh)/(OmBm)

where *R_h_* is the recalculated human residence time for an organ or tissue, *R_m_* is the originally calculated mouse residence time, *O_h_* is the human organ weight, *O_m_* is the mouse organ weight, *B_h_* is the human body weight, and *B_m_* is the mouse body weight.

Using OLINDA ver. 1.1 and a human adult as an anthropomorphic model, the organ or tissue absorbed doses for ^213^Bi and ^177^Lu were calculated using the recalculated human residence times obtained from the method stated above. For ^213^Bi, which has a branching decay chain, the contributions from daughter products 213Polonium (97.9%) and 209Thallium (2.1%) were summed with doses from ^213^Bi. In this calculation, the absorbed dose to normal organs and tissues in centigray per 37 megabecquerel administered (cGy/37 MBq) does not include any multiplier for quality factor or relative biological effectiveness for the alpha emissions from ^213^Bi and ^213^Po. However, the multiplier was used with the tumor as a target tissue. The tumor is not a target organ in the output results from OLINDA1.1, but it may be calculated separately using the same method as for the normal organs and tissues. For calculating tumor dose in units of centigray-equivalent per unit 37 MBq administered, all of the absorbed doses attributed to alpha emissions were multiplied by an arbitrary factor of 5 [17,18]. No such multiplier was used for calculating the absorbed dose to tumor tissue from ^177^Lu, which lacks alpha particles. To obtain the absorbed dose to tumor tissue for ^213^Bi in conventional units, one may divide the centigray-equivalent dose by a factor of five to yield cGy/37 MBq administered to obtain the absorbed dose in cGy/37 MBq.

Comparative RIT of B16-F10 melanoma tumor-bearing mice with ^177^Lu-h8C3 and ^213^Bi-h8C3. Female C57Bl6 mice were injected with 5 × 10^5^ B16-F10 melanoma cells into the right flank as described above. The mice were used for therapy when their tumors reached approximately 50 mm^3^. The mice were randomized into the group of five animals and treated with either 7.4 MBq ^213^Bi-h8C3, 14.8 MBq ^213^Bi-h8C3, 7.4 MBq ^177^Lu-h8C3, 14.8 MBq ^177^Lu-h8C3, or 80 μg unlabeled (“cold”) h8C3, or left untreated. Their tumors were measured every three days with electronic calipers to calculate the tumor volume for 21 days. The mice were weighted every 3 days. Their blood was analyzed on a weekly basis for white blood cells (WBC), red blood cells (RBC), and platelet (PLT) count. At the completion of the experiment mice were sacrificed and their blood was analyzed for ALT, AST, urea, and creatinine. Graphing and statistical analysis were completed using Prism GraphPad. Standard deviations were calculated from a minimum sample size of *n* = 3. Significance was calculated using the Student T-test with a *P*-value of less than 0.05 considered significant. 

## 3. Results

### 3.1. Humanized 8C3 mAb Specifically Localized in the Tumors, but not in Healthy Melanized Tissues 

We performed preliminary biodistribution of ^111^In-h8C3 in B16-F10 tumor-bearing mice and compared it to the previously reported by us biodistirubution of murine ^111^In-8C3 [12]. The biodistribution showed high accumulation of ^111^In-h8C3 with values being close to those of murine ^111^In-8C3 (Table S1) while the uptake in melanized healthy tissues such as eyes and skin on the tails was very minor (Figure S1). These results provided impetus for detailed biodistribution and microSPECT/CT imaging of ^111^In-h8C3 in B16-F10 melanoma-bearing mice. 

### 3.2. microSPECT/CT Imaging and Detailed Biodistribution Revealed Prolonged Retention of ^111^In-h8C3 in B16-F10 in the Tumors

**Figure 1:**
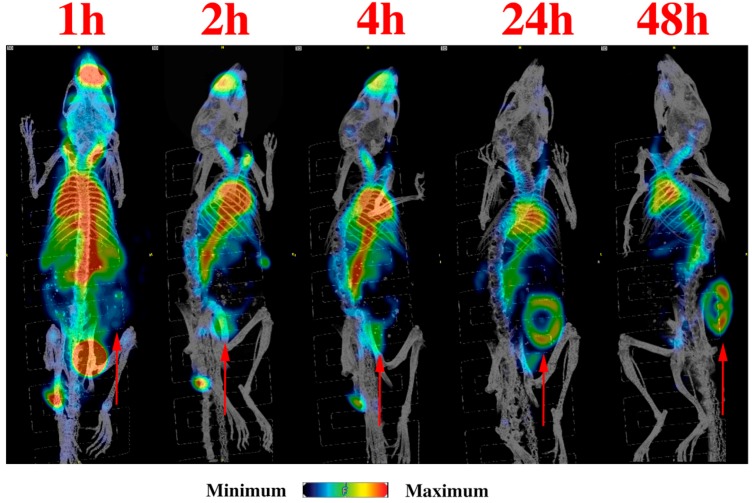
microSPECT/CT imaging of ^111^In-h8C3 in B16-F10 tumor-bearing mice. The red arrows indicate tumor location. Images presented are presented as maximum intensity projections (MIP) for clarity. Slice imaging of the 24 h time point is shown in Figure S2 to show tumor location more clearly.

**Figure 2:**
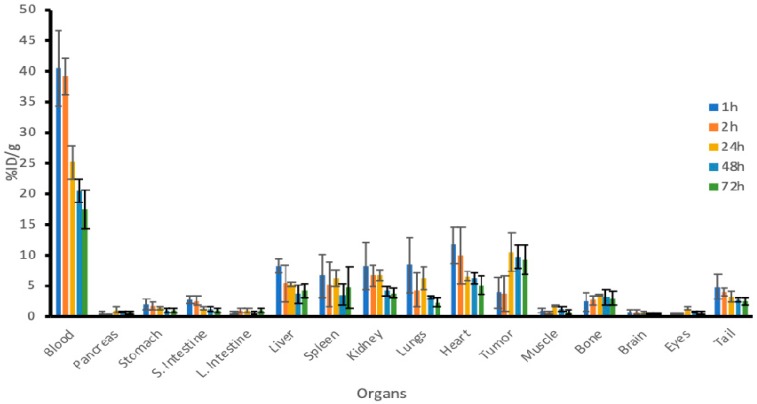
Detailed biodistribution of ^111^In-h8C3 in B16-F10 tumor-bearing mice.

microSPECT/CT imaging demonstrated that ^111^In-h8C3 started to accumulate in the tumor from the 1st hour post administration and was localized at the tumor site by 48 h (Figure 1). Our previous results show retention of the antibody in the tumor even at 216 h post injection [19]. The detailed biodistribution experiment which followed established that at 1 and 2 h post administration the uptake of ^111^In-h8C3 in B16-F10 tumors is approximately 4–5% ID/g, it reaches maximum value of 12% ID/g at 24 h and the slow wash-out phase starts at 48 h (Figure 2). There was no accumulation of the radiolabeled antibody in any organ including the melanized eyes and the tail skin of C57Bl6 mice. The results of the biodistribution were also used for mouse dosimetry calculations.

### 3.3. Mouse and Human Dosimetry Calculations Point at Differences Between ^177^Lu-h8C3 and ^213^Bi-h8C3 in Doses Delivered to the Tumor and Normal Organs

Mouse dosimetry calculations (Table 1) showed that ^177^Lu-h8C3 and ^213^Bi-h8C3 delivered very similar doses to all organs (*P* > 0.05). Antibodies radiolabeled with ^177^Lu via BCA CHX-A″-DTPA have high in vitro and in vivo stability and have been used in RIT [20,21]. The dose to the tumor, however, was almost 4 times higher for ^177^Lu-h8C3 (*P* = 0.01) which can be explained by the prolonged retention of the h8C3 mAb in the tumor and the relatively long physical half-life of ^177^Lu of 6.7 days. Extrapolation of the mouse dosimetry data to an adult human demonstrated that doses delivered to major organs and whole body by ^213^Bi-h8C3 (Table 2) will be approximately two times lower than those delivered by ^177^Lu-h8C3 (Table 2) while the doses to the tumor will be almost similar for both radionuclides.

### 3.4. ^213^Bi-h8C3 was More Effective and Safer than ^177^Lu-h8C3 in Slowing Down B16-F10 Tumor Growth

**Figure 3:**
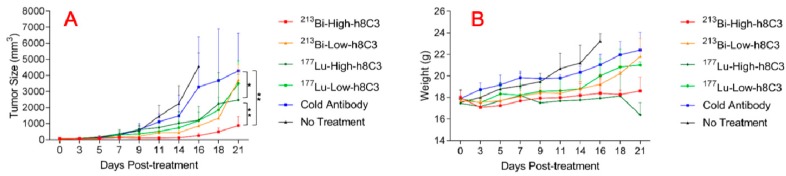
Tumor volumes and body weights of B16-F10 melanoma-bearing mice treated with ^177^Lu-h8C3 and ^213^Bi-h8C3. (**A**) Tumor volume and (**B**) body weight. Low dose—7.4 MBq; high dose—14.8 MBq. * indicate statistical significance.

**Figure 4:**
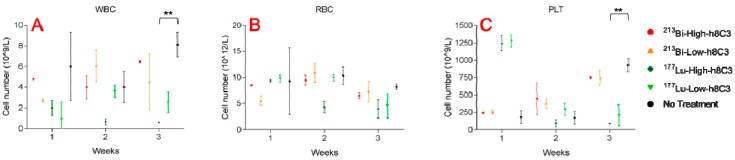
Blood chemistry (white blood cells, red blood cells and platelet) of B16-F10 melanoma-bearing mice treated with ^177^Lu-h8C3 and ^213^Bi-h8C3. (**A**–**C**)—WBC, RBC and PLT, respectively. Low dose—7.4 MBq; high dose—14.8 MBq. ** indicate statistical significance *P* = 0.001.

Figure 3A,B shows the tumor volume in mice with B16-F10 melanoma tumors after treatment with ^213^Bi-h8C3 and ^177^Lu-h8C3. While both radionuclide-labeled antibodies were able to considerably (*P* < 0.05) slow down the tumor growth, ^213^Bi-h8C3 was more effective than ^177^Lu-h8C3, and there was a pronounced dose response for ^213^Bi-h8C3, with 14.8 MBq (high dose) having more significant effect on the tumor volume than low dose 7.4 MBq (*P* = 0.01) (Figure 4A), while no dose response was observed for ^177^Lu-h8C3 (*P* = 0.5) (Figure 4A). The mice in the high dose groups had their body weight stable during the first half of the study but in the 2^nd^ part of the study the weight of mice in 14.8 MBq ^213^Bi-h8C3 group started to show an upward trend (Figure 4B), while the weight in 14.8 MBq ^177^Lu-h8C3 started to decline (Figure 4B). 

The blood chemistry which was evaluated at the end of weeks 1, 2, and 3 post RIT revealed that 14.8 MBq ^213^Bi-h8C3 did not cause any significant changes in WBC and RBC counts, and only a transient decrease in PLT count which normalized during week 3 (Figure 4A–C). In contrast, 14.8 MBq ^177^Lu-h8C3 had a pronounced and prolonged effect on significantly (*P* = 0.03) lowering WBC, RBC and PLT counts (Figure 4A–C). No kidney or liver toxicity was observed for both radionuclides when mice were sacrificed at the completion of the experiment. Overall, RIT of B16-F10 melanoma with ^213^Bi-h8C3 was more effective and safe than with ^177^Lu-h8C3.

## 4. Discussion

In this study we performed comparative radioimmunotherapy (RIT) of experimental B16-F10 melanoma with novel humanized IgG to melanin h8C3 labeled with a beta emitter ^177^Lu and an alpha-emitter ^213^Bi, as well as biodistribution, microSPECT/CT imaging, and mouse and human dosimetry calculations. The results of the study demonstrate that ^213^Bi-h8C3 was more effective and safe than with ^177^Lu-h8C3 in a very aggressive murine melanoma model. Previously, we suggested targeting melanin, an intracellular antigen which becomes available for the antibody binding in the leaky or dying tumor cells, with a radiolabeled murine IgM pre-clinically and in Phase I clinical trial in patients with metastatic melanoma [22,23]. However, the IgMs are difficult to produce under the cGMP (current Good Manufacturing Practice) conditions, to purify and to radiolabel, as well as to humanize. The continuous development of RIT targeting melanin towards the clinical product calls for a humanized IgG isotype which can be administer to patients several times without precipitating immune side effects. For this reason, when an IgG to melanin became available [24], we performed its evaluation as a RIT reagent in B16-F10 murine melanoma and upon obtaining encouraging results [12], carried out its humanization, resulting in a humanized IgG h8C3. Though the previous Phase I study of murine melanin-binding IgM was performed with a beta-emitter ^188^Re, we made a decision to proceed with a trivalent radiometal such as ^213^Bi or ^177^Lu because initial attempts at using ^188^Re proved difficult as the humanized 8C3 antibody was not amenable to the harsh conditions needed for ^188^Re labeling [19]. In addition, in a decade since a Phase I trial was conducted, it became clear that alpha-emitters can be effective in treating solid tumors not only micrometastatic disease as it was thought before [25,26,27,28,29].

Promising results from our initial and in-depth biodistributions showed high tumor uptake, and, very importantly, showed no uptake in the skin of the tail or eyes of the mice. As both the skin and eyes contain high amounts of intracellular melanin in C57BL6 mice, absence of uptake in these organs confirms the non-internalizing nature of h8C3 mAb and its inability to reach melanin in intact cells. We then performed a side by side comparison of the short-lived alpha-emitter ^213^Bi (half-life 46 min), and the long-lived beta-emitter ^177^Lu (half-life 6.7 days). Both of these radionuclides have been used in a multitude of clinical trials [25]. In addition, ^177^Lu-labeled peptide (Lutathera) is clinically approved for treatment of somatostatin-positive neuroendocrine tumors. Dosimetry calculations in mice predicted that ^177^Lu-h8C3 would deliver a four-times higher radiation dose to the tumor than ^213^Bi-h8C3. However, our results indicate that ^213^Bi was a much more effective radionuclide for melanin targeted RIT. One possible explanation for this striking difference in efficacy could be the ability of the short lived ^213^Bi nuclide to deliver its radiation dose in a short period of time; thereby allowing its intense, high relative biological effectiveness (RBE) to counteract the aggressive growth of B16-F10. In addition, alpha radiation is capable of initiating other radiobiological effects such cell cycle arrest and “bystander” effect [26,27] which could have contributed to the superior efficacy of ^213^Bi-h8C3. Interestingly, the pre-clinical and clinical studies in other types of cancer, where ^213^Bi- and ^177^Lu-labeled targeting molecules were compared side by side, also demonstrated more pronounced effect of ^213^Bi radiolabel on the tumor [28,29,30].

## 5. Conclusions

^213^Bi-labeled humanized IgG to melanin h8C3 proved to be effective and safe in treatment of aggressive B16-F10 melanoma. The experiments on multiple dosing of RIT with this agent as well as on combining it with immunotherapy are currently on-going.

## Figures and Tables

**Table 1:** Doses to the normal organs and melanoma tumors in mice delivered by ^177^Lu-h8C3 and ^213^Bi-h8C3.

Target Organ	Bismuth-213		Lutetium-177	
	Absorbed Dose (cGy/37 kBq admin.)	Energy Absorbed Fraction	Absorbed Dose (cGy/37 kBq admin.)	Energy Absorbed Fraction
Blood	8.590	1.0	6.440	0.95
Pancreas	0.099	1.0	0.177	0.85
Stomach	0.389	1.0	0.346	0.96
Small Intestine	0.548	1.0	0.301	0.90
Large intestine	0.116	1.0	0.386	0.90
Liver	1.800	1.0	1.330	0.88
Spleen	1.409	1.0	1.707	0.87
Kidney	1.732	1.0	1.550	0.93
Lungs	2.010	1.0	1.079	0.89
Heart	2.453	1.0	1.822	0.93
Tumor	0.805	1.0	3.429	0.89
Muscle	0.158	1.0	0.298	0.99
Bone	0.502	1.0	0.679	0.50
Brain	0.113	1.0	0.159	0.95
Eyes	0.108	1.0	0.146	0.60
Tail	1.014	1.0	0.917	0.97

**Table 2:** Absorbed doses in cGy/37 MBq to the normal organs and melanoma tumors in an adult man delivered by ^213^Bi and ^177^Lu. A Centigray-equivalent dose per 37 MBq administered, alpha multiplier = 5 was applied for ^213^Bi tumor dose calculation. Complete dose tables can be found in the supplementary (Tables S2 and S3).

Target Organ	Total ^213^Bi Dose	Total ^177^Lu Dose
Adrenals	8.70 × 10^−2^	2.48 × 10^−1^
Brain	3.15 × 10^−3^	2.29 × 10^−2^
Breasts	8.65 × 10^−2^	2.38 × 10^−1^
Gallbladder Wall	8.72 × 10^−2^	2.50 × 10^−1^
Lower Large Intestine Wall	8.74 × 10^−2^	2.53 × 10^−1^
Small Intestine	8.76 × 10^−2^	2.56 × 10^−1^
Stomach Wall	8.72 × 10^−2^	2.48 × 10^−1^
Upper Large Intestine Wall	8.75 × 10^−2^	2.54 × 10^−1^
Heart Wall	6.74 × 10^−3^	3.65 × 10^−2^
Kidneys	2.36 × 10^−2^	9.00 × 10^−2^
Liver	5.68 × 10^−2^	1.69 × 10^−1^
Lungs	7.26 × 10^−3^	3.02 × 10^−2^
Muscle	3.94 × 10^−3^	3.51 × 10^−2^
Ovaries	8.75 × 10^−2^	2.54 × 10^−1^
Pancreas	1.76 × 10^−3^	2.59 × 10^−2^
Red Marrow	1.15 × 10^−1^	1.88 × 10^−1^
Osteogenic Cells	8.30 × 10^−1^	7.56 × 10^−1^
Skin	8.62 × 10^−2^	2.34 × 10^−1^
Spleen	2.91 × 10^−3^	2.68 × 10^−2^
Testes	8.68 × 10^−2^	2.42 × 10^−1^
Thymus	8.68 × 10^−2^	2.44 × 10^−1^
Thyroid	8.68 × 10^−2^	2.44 × 10^−1^
Urinary Bladder Wall	8.73 × 10^−2^	2.50 × 10^−1^
Uterus	8.75 × 10^−2^	2.55 × 10^−1^
Total Body	8.93 × 10^−2^	2.53 × 10^−1^
Tumor	2.98 × 10^−1^	3.36 × 10^−1^

## Supplementary Materials

The following are available online at https://www.mdpi.com/1999-4923/11/7/348/s1. Table S1: Statistical comparison of antibody uptake between mouse and humanized 8C3 antibody, Table S2: Absorbed doses in cGy/37 MBq to the normal organs and melanoma tumors in an adult man delivered by ^213^Bi and its daughters, Table S3: Absorbed doses in cGy/37 MBq to the normal organs and melanoma tumors in an adult man delivered by 177Lu, Figure S1: Preliminary biodistribution of ^111^In-h8C3 in B16-F10 tumor-bearing mice, Figure S2: microSPECT/CT Slice imaging of ^111^In-h8C3 in B16-F10 tumor-bearing mouse at 24 h post injection.
