# Supplementary material for: Comparative Radioimmunotherapy of Experimental Melanoma with Novel Humanized Antibody to Melanin Labeled with 213Bismuth and 177Lutetium
Source: Pharmaceutics. 2019 Jul 18;11(7):348. doi: 10.3390/pharmaceutics11070348 (PMC6680821; doi:10.3390/pharmaceutics11070348)
Supplement: Supplementary file 1 [file pharmaceutics-11-00348-s001.pdf]

Supplementary Materials: Comparative Radioimmunotherapy of Experimental Melanoma with Novel Humanized Antibody to Melanin Labeled with 213Bismuth and 177Lutetium

Kevin J. H. Allen, Rubin Jiao, Mackenzie E. Malo, Connor Frank, Darrell R. Fisher, David Rickles and Ekaterina Dadachova

Table S1. Statistical comparison of antibody uptake between mouse and humanized 8C3 antibody.

Organ	Mouse Ab %ID/g	Human Ab %ID/G	p value	stat
Blood	31.3933519	24.118107	0.0391	*
Tumor	15.193809	10.6030806	0.4419	ns
Spleen	9.51605417	7.21729285	0.2564	ns
Kidneys	7.67318241	5.77245188	0.2271	ns
Liver	10.1497756	8.05200569	0.3093	ns
Brain	0.44544925	0.42564811	0.9893	ns
Lungs	11.4052144	6.72586198	0.1085	ns
Stomach	1.82375371	1.31886843	0.1609	ns
Small Ints	3.26209637	2.43430403	0.0129	*
Large Ints	2.10842766	1.47324529	0.1331	ns
Thigh Muscle	1.92528808	1.70014015	0.4481	ns
Femur	3.14234705	2.95363782	0.8756	ns
Eye	1.06341217	0.81492002	0.3639	ns
Tail	4.24436179	3.34845973	0.8023	ns
Heart	9.49981318	9.08266293	0.7636	ns

Table S2. Absorbed doses in cGy/37 MBq to the normal organs and melanoma tumors in an adult man delivered by ^{213}Bi and its daughters.

Target Organ	Alpha	Beta	Photon	Total	(Numeric)
Adrenals	7.19×10^{-2}	1.34×10^{-2}	1.77×10^{-3}	8.70×10^{-2}	0.087
Brain	2.08×10^{-3}	3.87×10^{-4}	6.90×10^{-4}	3.15×10^{-3}	0.00315
Breasts	7.19×10^{-2}	1.34×10^{-2}	1.27×10^{-3}	8.65×10^{-2}	0.0865
Gallbladder Wall	7.19×10^{-2}	1.34×10^{-2}	1.97×10^{-3}	8.72×10^{-2}	0.0872
Lower Large Intestine Wall	7.19×10^{-2}	1.34×10^{-2}	2.16×10^{-3}	8.74×10^{-2}	0.0874
Small Intestine	7.19×10^{-2}	1.34×10^{-2}	2.40×10^{-3}	8.76×10^{-2}	0.0876
Stomach Wall	7.19×10^{-2}	1.34×10^{-2}	1.93×10^{-3}	8.72×10^{-2}	0.0872
Upper Large Intestine Wall	7.19×10^{-2}	1.34×10^{-2}	2.29×10^{-3}	8.75×10^{-2}	0.0875
Heart Wall	4.35×10^{-2}	8.09×10^{-2}	1.57×10^{-3}	6.74×10^{-3}	0.00674
Kidneys	1.88×10^{-2}	3.46×10^{-3}	1.41×10^{-3}	2.36×10^{-2}	0.0236
Liver	4.67×10^{-2}	8.68×10^{-3}	1.40×10^{-3}	5.68×10^{-2}	0.0568
Lungs	5.09×10^{-3}	9.47×10^{-4}	1.22×10^{-3}	7.26×10^{-3}	0.00726
Muscle	2.16×10^{-3}	4.02×10^{-4}	1.38×10^{-3}	3.94×10^{-3}	0.00394
Ovaries	7.19×10^{-2}	1.34×10^{-2}	2.22×10^{-3}	8.75×10^{-2}	0.0875
Pancreas	1.08×10^{-4}	1.99×10^{-5}	1.63×10^{-3}	1.76×10^{-3}	0.00176
Red Marrow	1.04×10^{-1}	9.44×10^{-3}	1.72×10^{-3}	1.15×10^{-1}	0.115
Osteogenic Cells	8.05×10^{-1}	2.31×10^{-2}	2.10×10^{-3}	8.30×10^{-1}	0.830
Skin	7.19×10^{-2}	1.34×10^{-2}	9.55×10^{-4}	8.62×10^{-2}	0.0862
Spleen	1.39×10^{-3}	2.57×10^{-4}	1.27×10^{-3}	2.91×10^{-3}	0.00291
Testes	7.19×10^{-2}	1.34×10^{-2}	1.53×10^{-3}	8.68×10^{-2}	0.0868
Thymus	7.19×10^{-2}	1.34×10^{-2}	1.55×10^{-3}	8.68×10^{-2}	0.0868
Thyroid	7.19×10^{-2}	1.3×10^{-2}	1.55×10^{-3}	8.68×10^{-2}	0.0868
Urinary Bladder Wall	7.19×10^{-2}	1.34×10^{-2}	2.05×10^{-3}	8.73×10^{-2}	0.0873
Uterus	7.19×10^{-2}	1.34×10^{-2}	2.30×10^{-3}	8.75×10^{-2}	0.0875
Total Body	7.41×10^{-2}	1.38×10^{-2}	1.42×10^{-3}	8.93×10^{-2}	0.0893
Centigray-equivalent dose per 37 MBq administered, alpha multiplier = 5					
Tumor	2.93×10^{-1}	3.02×10^{-3}	1.32×10^{-3}	2.98×10^{-1}	0.298

Table S3. Absorbed doses in cGy/37 MBq to the normal organs and melanoma tumors in an adult man delivered by ^{177}Lu.

Target Organ	Beta	Photon	Total	(Numeric)
Adrenals	2.21×10^{-1}	2.69×10^{-2}	2.48×10^{-1}	0.248
Brain	1.17×10^{-2}	1.13×10^{-2}	2.29×10^{-2}	0.023
Breasts	2.21×10^{-1}	1.65×10^{-2}	2.38×10^{-1}	0.238
Gallbladder Wall	2.21×10^{-1}	2.86×10^{-2}	2.50×10^{-1}	0.250
Lower Large Intestine Wall	2.21×10^{-1}	3.17×10^{-2}	2.53×10^{-1}	0.253
Small Intestine	2.21×10^{-1}	3.51×10^{-2}	2.56×10^{-1}	0.256
Stomach Wall	2.21×10^{-1}	2.71×10^{-2}	2.48×10^{-1}	0.248
Upper Large Intestine Wall	2.21×10^{-1}	3.32×10^{-2}	2.54×10^{-1}	0.254
Heart Wall	1.33×10^{-2}	2.32×10^{-2}	3.65×10^{-2}	0.037
Kidneys	6.90×10^{-2}	2.10×10^{-2}	9.00×10^{-2}	0.090
Liver	1.49×10^{-1}	2.05×10^{-2}	1.69×10^{-1}	0.169
Lungs	1.18×10^{-2}	1.84×10^{-2}	3.02×10^{-2}	0.0302
Muscle	1.58×10^{-2}	1.93×10^{-2}	3.51×10^{-2}	0.0351
Ovaries	2.21×10^{-1}	3.32×10^{-2}	2.54×10^{-1}	0.254
Pancreas	1.09×10^{-3}	2.48×10^{-2}	2.59×10^{-2}	0.0259
Red Marrow	1.64×10^{-1}	2.38×10^{-2}	1.88×10^{-1}	0.188
Osteogenic Cells	7.12×10^{-1}	4.43×10^{-2}	7.56×10^{-1}	0.756
Skin	2.21×10^{-1}	1.25×10^{-2}	2.34×10^{-1}	0.234
Spleen	7.90×10^{-3}	1.89×10^{-2}	2.68×10^{-2}	0.0268
Testes	2.21×10^{-1}	2.11×10^{-2}	2.42×10^{-1}	0.242
Thymus	2.21×10^{-1}	2.26×10^{-2}	2.44×10^{-1}	0.244
Thyroid	2.21×10^{-1}	2.30×10^{-2}	2.44×10^{-1}	0.244
Urinary Bladder Wall	2.21×10^{-1}	2.92×10^{-2}	2.50×10^{-1}	0.250
Uterus	2.21×10^{-1}	3.39×10^{-2}	2.55×10^{-1}	0.255
Total Body	2.32×10^{-1}	2.16×10^{-2}	2.53×10^{-1}	0.253
Tumor	3.14×10^{-1}	2.23×10^{-2}	3.36×10^{-1}	0.336

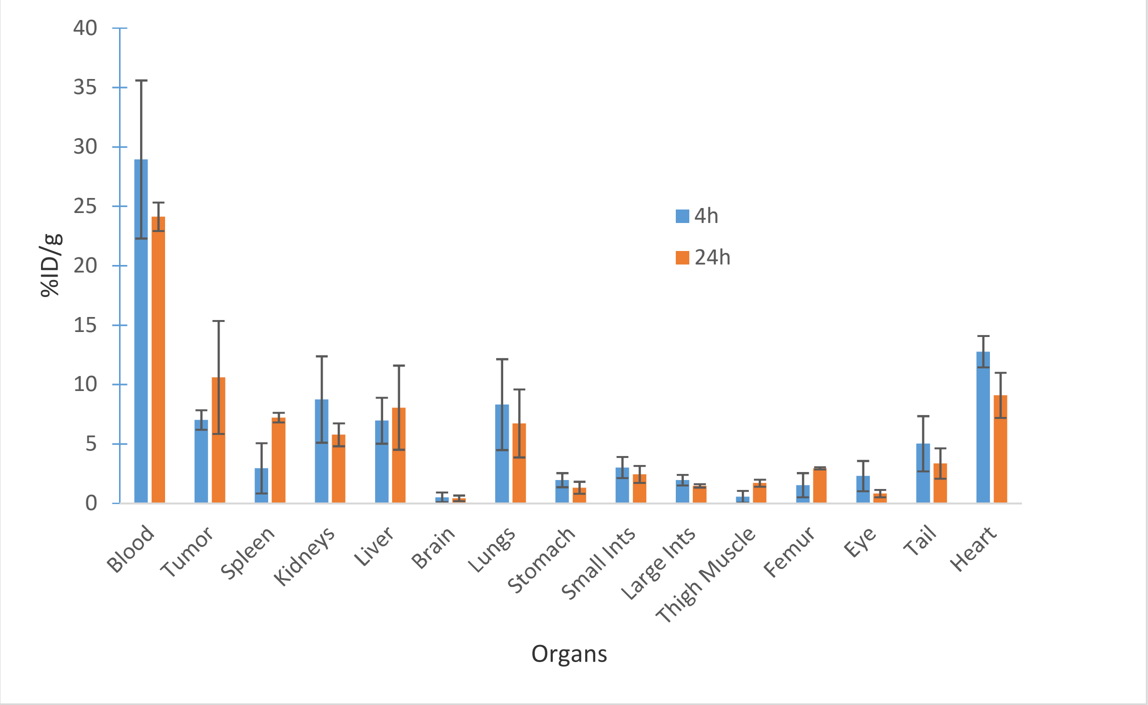

Figure S1. Preliminary biodistribution of ^{111}In-h8C3 in B16-F10 tumor-bearing mice.

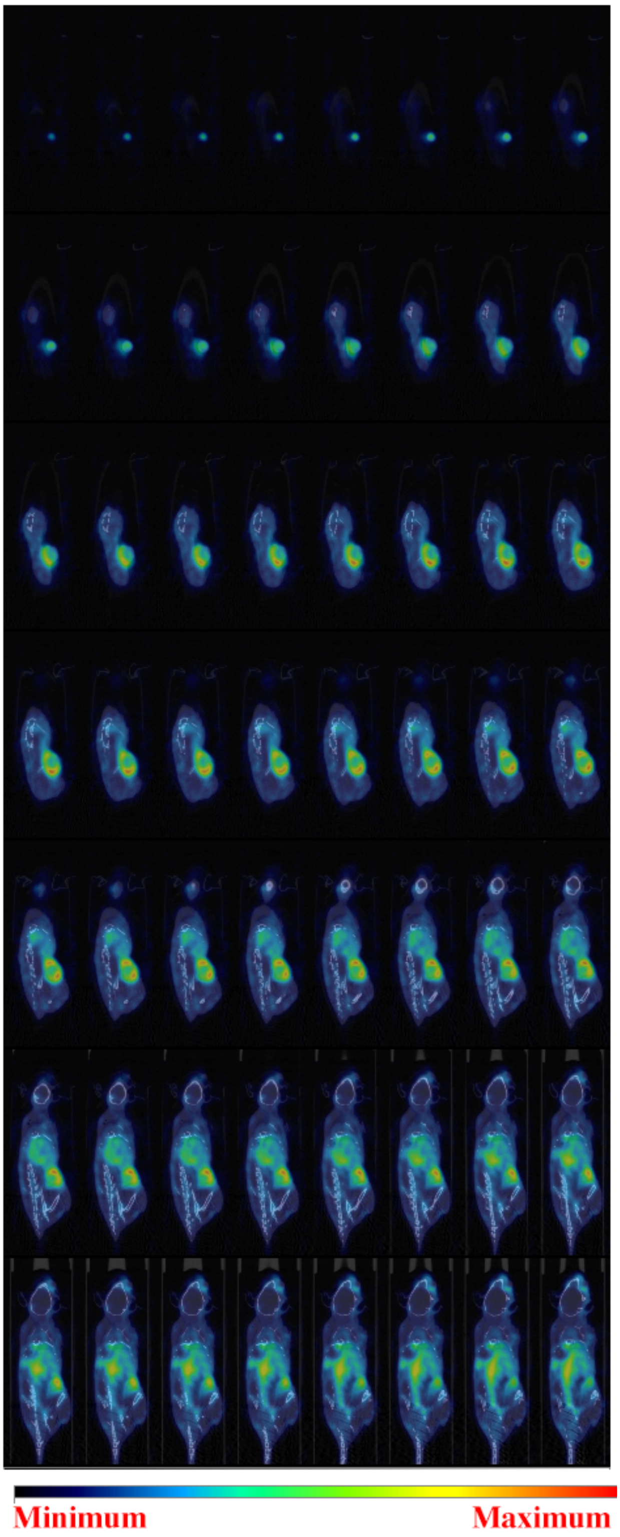

Figure S2. microSPECT/CT Slice imaging of ^{111}In-h8C3 in B16-F10 tumor-bearing mouse at 24 h post injection.
